# Preemptive noradrenaline infusion: a game-changer against post-induction hypotension in surgical sepsis: a randomized double blind trial

**DOI:** 10.1186/s12871-026-03783-2

**Published:** 2026-04-11

**Authors:** Mina Adolf Helmy, Mohamed Saber Mostafa, Arsany Talaat Saber, Mai A. Ali, Ismail A. Ismail, Beshoy Atef Naguib, Mohamed Maher Elaraby, Lydia Magdy Milad

**Affiliations:** 1 Department of Anesthesia and Critical Care Medicine, Cairo University, Cairo, Egypt; 2 Department of General Surgery, Cairo University, Cairo, Egypt; 3 Lecturer of Anesthesia and Critical Care Medicine, Faculty of Medicine, Cairo University, Cairo, Egypt

**Keywords:** Hypotension, Sepsis, Acute kidney injury

## Abstract

**Background:**

Post-induction hypotension is a frequent and clinically significant complication in patients with sepsis undergoing source control surgery, often leading to adverse outcomes such as acute kidney injury. Despite its prevalence, targeted strategies to prevent post-induction hypotension in this population remain underexplored. We aimed to evaluate the efficacy of prophylactic noradrenaline infusion (0.2 µg/kg/min) in reducing the incidence of post-induction hypotension in patients with surgical sepsis.

**Methods:**

In this randomized controlled trial, 80 adult patients with surgical sepsis were allocated to receive either noradrenaline infusion or saline placebo before anesthesia induction. Baseline demographics, laboratory values, and intraoperative parameters were also recorded. The primary outcome was the incidence of postinduction hypotension, defined as a > 20% reduction in mean arterial pressure within 10 min of induction. The secondary outcomes included the time to the first hypotensive episode, ephedrine requirements, incidence of acute kidney injury, and other postoperative complications.

**Results:**

The baseline characteristics were comparable between the groups. The noradrenaline group demonstrated a significantly lower incidence of post-induction hypotension (10% vs. 45%, *p* < 0.001). Moreover, the incidence of acute kidney injury was markedly lower in the noradrenaline-treated cohort (5%) than in the saline-control group (23%). In contrast, no statistically significant differences were observed between the groups with respect to other clinical outcomes, including myocardial infarction, renal replacement therapy, stroke, and delirium.

**Conclusion:**

Prophylactic noradrenaline infusion before anesthesia induction significantly improves hemodynamic stability and reduces the risk of acute kidney injury in patients with sepsis undergoing source control surgery. These findings support the use of this technique as a safe and effective strategy for preventing post-induction hypotension in high-risk surgical populations.

**Trial registration:**

The clinical study was registered on ClinicalTrials.gov by the principal investigator, M. Helmy, under the identifier NCT07022730 on June 7, 2025.

## Highlights

Prophylactic noradrenaline infusion (0.2 µg/kg/min) significantly reduced the incidence and severity of post-induction hypotension in septic patients undergoing source control surgery.

Patients receiving noradrenaline experienced fewer hypotensive episodes, delayed onset of hypotension, and required less ephedrine to maintain target blood pressure.

The intervention was associated with a markedly lower rate of acute kidney injury (5% vs. 23%), suggesting improved organ perfusion and protective effects during anesthesia induction.

## Introduction

Post‑induction hypotension (PIH) is a frequent and clinically significant complication of general anesthesia, occurring in nearly half of surgical patients. It is typically defined as a reduction in mean arterial pressure (MAP) exceeding 20% from baseline values, and its occurrence has been consistently associated with adverse perioperative outcomes [1, 2]. PIH is particularly concerning in patients with sepsis, who already suffer from impaired vascular tone, systemic inflammation, and altered myocardial function [3, 4]. In this vulnerable population, even transient hypotension can precipitate organ hypoperfusion, leading to acute kidney injury (AKI), myocardial ischemia, and increased postoperative morbidity [5–7]. Despite its prevalence and impact, PIH remains under‑recognized, and strategies for its prevention are not yet standardized across clinical practice.

Several patient‑related risk factors have been identified for PIH, including advanced age, elevated body weight, and preoperative hypertension [8, 9]. Mechanistically, PIH may result from myocardial depression, reduced systemic vascular resistance, or a combination of both [1]. In septic patients, these mechanisms are exacerbated by profound vasodilation and impaired vascular responsiveness, making them especially prone to hemodynamic instability during anesthetic induction. Prior studies have demonstrated that early recognition and proactive management of PIH are essential to prevent downstream complications [10–12]. However, most existing evidence has focused on general surgical populations, with limited attention to patients undergoing source control surgery for sepsis, a group in whom PIH may carry particularly severe consequences.

Pharmacologic prophylaxis has emerged as a promising approach to mitigate PIH. Vasopressors such as phenylephrine have been studied in elective surgical settings, with evidence suggesting that pre‑induction administration can reduce the incidence of hypotension [13]. In cardiac anesthesia, prophylactic noradrenaline infusion has been shown to maintain vascular tone and prevent hypotensive episodes during high‑risk procedures such as transcatheter aortic valve implantation [14]. Noradrenaline, a potent α‑adrenergic agonist with mild β‑activity, counteracts sepsis‑related vasodilation and supports systemic vascular resistance. These properties make it a theoretically attractive agent for preventing PIH in septic patients, yet no randomized controlled trial has specifically evaluated its prophylactic use in this context.

Given the high incidence of PIH in sepsis and its association with adverse outcomes, there is a pressing need to explore effective preventive strategies. To address this gap, we conducted a randomized, double‑blind controlled trial to investigate the efficacy of pre‑induction noradrenaline infusion in reducing PIH among patients with surgical sepsis. We hypothesized that prophylactic administration of noradrenaline would improve peri‑induction hemodynamic stability, reduce the incidence and severity of hypotension, and potentially mitigate secondary complications such as AKI. By focusing on this high‑risk population, our study aims to provide evidence for a simple, reproducible intervention that could enhance perioperative safety in sepsis management.

## Methods

This randomized, double‑blind controlled trial was conducted at Cairo University Hospital following approval from the Cairo University Faculty of Medicine Research Ethics Committee (N‑173‑2025) and registration at clinicaltrials.gov (NCT07022730). The study adhered to CONSORT standards. Eighty adult patients diagnosed with surgical sepsis, defined as an increase in sequential organ failure assessment (SOFA) score by two or more points and requiring source control surgery under general anesthesia, were enrolled between June 2025 and September 2025 and randomly assigned to either a control group (*n* = 40) or a noradrenaline group (*n* = 40).

### Randomization and blinding

Participants were randomized using a computer-generated allocation sequence with permuted blocks of four to ensure balanced group assignment. Allocation concealment was maintained using sequentially numbered, sealed, opaque envelopes prepared by an independent research assistant who had no role in patient recruitment, clinical care, or data collection. The attending anesthesiologist screened and enrolled eligible participants. To ensure double blinding, an independent pharmacist prepared the study infusions (noradrenaline or normal saline) in identical syringes that were indistinguishable in appearance and labeling. These syringes were administered by clinical staff who were unaware of group assignments. Throughout the study period, patients, anesthesiologists administering the intervention, perioperative healthcare providers, outcome assessors, and data analysts all remained blinded to the treatment allocation.

The inclusion criteria comprised hemodynamically stable patients at baseline (MAP ≥ 65 mmHg), while the exclusion criteria included pre-existing cardiac arrhythmias, left ventricular ejection fraction below 45%, renal impairment, defined as baseline serum creatinine > 1.5 × Upper normal limit (UNL) (> 1.8 mg/dL male; >1.5 mg/dL female) however patients with mildly elevated baseline serum creatinine (above UNL but below 1.5 × UNL) were eligible for inclusion., pregnancy, and the need for rapid sequence induction. All patients received initial management in the emergency department according to the Sepsis-3 guidelines and the one-hour bundle. In the noradrenaline group, Norepinephrine was administered using Norepinephrine‑Mirola (INAD Pharma for Mirola, Cairo, Egypt). Each 4 ml ampoule contains norepinephrine bitartrate 8 mg, equivalent to 4 mg norepinephrine base. In accordance with standard pharmacological practice, norepinephrine is supplied as the tartrate salt, and dosing is reported as base equivalence at a fixed rate of 0.2 µg/kg/min, expressed as norepinephrine base, to ensure transparency and comparability with other clinical investigations [15, 16]. via a continuous infusion initiated five minutes before induction and maintained for 10 min after induction. The control group received a placebo normal saline infusion under identical conditions to preserve blinding. In both groups, infusions were stopped 10 min after induction and syringes discarded.

Notably, noradrenaline infusion was initiated 5 min before induction and continued until 10 min after induction, for a total duration of 15 min.

Study infusions (noradrenaline or saline) were administered through a dedicated peripheral intravenous line using a large-bore cannula placed before induction. This line was reserved exclusively for the study drug to maintain blinding and prevent admixture with other agents. Central venous access was not routinely established before induction.

After confirming fasting hours, standard monitoring, including noninvasive blood pressure, electrocardiography, and pulse oximetry, was performed. Anesthesia was induced with intravenous fentanyl (2 µg/kg), followed by Ketofol (1:1 ketamine: propofol) administered in 10 mg increments until loss of verbal response, and atracurium (0.5 mg/kg IV). Patients were preoxygenated with 100% oxygen for two minutes and ventilated with 100% oxygen for four minutes post-induction before intubation, in accordance with institutional protocol to ensure adequate oxygenation and hemodynamic stability. Mechanical ventilation was set at a tidal volume of 6 mL/kg and a respiratory rate of 16 breaths/min, targeting an end-tidal CO₂ of 30 mmHg to avoid hypercapnia in septic patients.

Intraoperative fluid therapy consisted of lactated Ringer’s solution at 4 mL/kg, with blood loss replaced at a 3:1 ratio. Hemodynamic parameters, including noninvasive blood pressure and heart rate, were recorded before, immediately after, and at 1-minute intervals for 10 min post-induction, and every 3 min until the end of surgery. Hypotension was defined as a ≥ 20% reduction in MAP from baseline occurring within the first 10 min after induction, and ephedrine was administered in 6 mg increments to maintain MAP within 20% of baseline. Atropine (0.5 mg IV) was administered if hypotension was accompanied by bradycardia (HR < 60 bpm).

After the 10‑minute post‑induction period, hypotensive episodes were managed with ephedrine boluses of 6 mg, administered as required up to a cumulative maximum dose of 60 mg. In cases where hypotension persisted despite the maximum ephedrine administration, noradrenaline infusion was commenced using a new syringe and titrated to achieve and maintain adequate hemodynamic stability.

### Data collection and study outcomes

The collected data included demographic variables (age, sex, BMI, SOFA score, source of sepsis, and duration of surgery), hemodynamic measurements, and laboratory values, particularly serum creatinine, which was monitored daily to assess acute kidney injury (AKI). In addition, the number of patients with serum creatinine values exceeding the upper normal limit (UNL; >1.2 mg/dL for males and > 1.0 mg/dL for females) was recorded.

The primary outcome was the incidence of PIH, defined as a ≥ 20% reduction in MAP from the baseline value within 10 min after induction. Baseline MAP was calculated as the average of three consecutive measurements obtained before starting the infusion. Secondary outcomes included the incidence of severe hypotension, defined as ≥ 40% reduction in MAP from the baseline value, the number of hypotensive episodes, and ephedrine requirements. Exploratory outcomes included the incidence of postoperative myocardial infarction (defined by peak troponin T ≥ 99th percentile upper reference limit within 7 days), acute kidney injury (AKI; defined as the highest postoperative value > 1.5-fold or 0.3 mg/dL greater than the preoperative value), incidence of stroke (confirmed by neurological deficits and CT imaging), incidence of delirium (assessed via CAM-ICU), number of patients requiring noradrenaline infusion after 10 min postinduction window, percentage of increase in serum creatinine, calculated as maximum postoperative creatinine within 7 days minus preoperative creatinine divided by preoperative creatinine and expressed as percentage, and the need for renal replacement therapy (RRT).

### Sample size and statistical methods

The sample size was determined using G*Power software version 3.1.9.7 (Heinrich Heine University Düsseldorf, Germany). A pilot study involving 10 patients revealed that seven developed PIH in the context of sepsis. Assuming a 50% reduction in PIH incidence is clinically significant and setting the study power at 80% with an alpha error of 0.05, the minimum required sample size was calculated to be 74 patients. To account for potential dropouts, the sample size was increased to 80, with 40 patients in each group.

The Shapiro-Wilk test was used to evaluate the normality of the data. Continuous variables are reported as either the median with interquartile range or the mean ± standard deviation, depending on the data distribution. The Mann–Whitney U test was used to compare non-normally distributed continuous variables. Categorical variables were summarized as counts and percentages and analyzed using the chi-squared test or Fisher’s exact test, as appropriate. Serial changes in MAP were evaluated using a two-way ANOVA to examine the time and group effects. The time to the first hypotensive episode was analyzed using Kaplan–Meier survival analysis, and the differences between groups were assessed using the log-rank test. Secondary outcomes were grouped into predefined families to account for multiple comparisons, and Bonferroni correction was applied within each family using the direct adjustment method. Specifically, raw p-values were multiplied by the number of outcomes in the corresponding family. The hemodynamic family included five outcomes (incidence of severe hypotension, number of hypotensive episodes, time to first hypotensive episode, need for additional noradrenaline, and ephedrine requirements). The postoperative family comprised six outcomes (incidence of AKI, need for RRT, incidence of delirium, incidence of MI, maximum change in creatinine, and incidence of stroke). The primary outcome was analyzed without multiplicity correction. Both unadjusted and adjusted p-values for secondary outcomes are reported side by side and interpreted cautiously as exploratory findings.

All statistical analyses were performed using MedCalc software, version 19 (Mariakerke, Belgium) and SPSS (version 26) for Microsoft Windows (Armonk, NY: IBM Corp.)

## Results

**Fig. 1:**
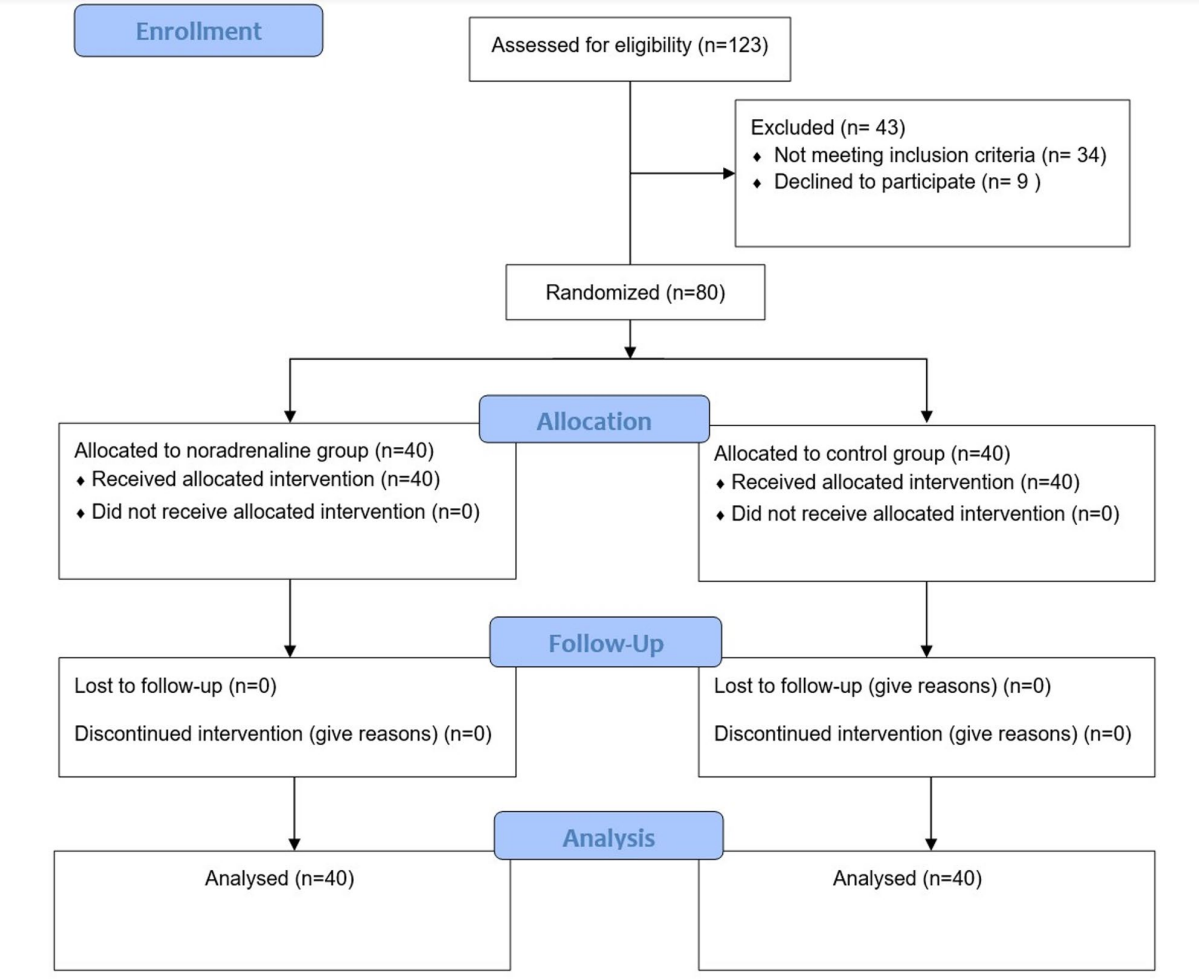
Patient enrollment

Of the 123 patients screened for eligibility, 43 were excluded, and 80 were ultimately included in the final analysis. Reasons for exclusion were as follows: 9 patients declined to participate, 12 had hemodynamic instability before induction, 10 had preoperative renal impairment (defined as baseline serum creatinine > 1.5 × the UNL; i.e., > 1.8 mg/dL in males and > 1.5 mg/dL in females), 8 required rapid sequence induction, and 4 had poor systolic function (ejection fraction < 45%) (Fig. 1).

Baseline demographic and clinical characteristics were comparable between the noradrenaline and control groups, with no statistically significant differences observed in age, sex distribution, body weight, height, BMI, SOFA scores, baseline heart rate, or mean blood pressure. Comorbidities, including diabetes mellitus, hypertension, and chronic obstructive pulmonary disease (COPD), and others, were similarly distributed across both groups. Additionally, the sources of sepsis and intraoperative variables, such as procedure duration, estimated blood loss, and volume of fluid administration, did not differ significantly (Table 1).

**Table 1:** Preoperative and baseline characteristics, data presented as mean$\:\pm\:$standard deviation, median (quartiles), and count (frequency)

	Noradrenaline group (*n* = 40)	Control group (*n* = 40)	*P* value
Age (years)	51(32–61)	48(38–57)	0.769
Male sex	24/40 (60%)	25/40 (63%)	> 0.999
Weight (Kg)	78.6$\:\pm$14.3	79.5$\:\pm\:$10.8	0.771
Height (m)	1.65$\:\pm\:$ 0.06	1.65$\:\pm\:$0.05	0.764
BMI (Kg/m^2^)	28.6$\:\pm\:$4.2	29.1$\:\pm\:$3.5	0.550
SOFA score	4(2–5)	3(3–4)	0.798
Baseline HR (beats/min)	99$\:\pm\:$14	97$\:\pm\:$13	0.613
Baseline MAP (mmHg)	72$\:\pm\:$ 8	71$\:\pm\:$8	0.691
Comorbidity			
DM	17/40 (43%)	19/40 (48%)	0.653
HTN	15/40 (38%)	15/40 (38%)	> 0.999
SLE	2/40 (5%)	1/40 (2.5%)	> 0.999
COPD	5/40 (13%)	3/40 (8%)	0.712
BA	3/40 (8%)	3/40 (8%)	> 0.999
Hypothyroidism	0/40 (0%)	2/40 (5%)	0.494
Source of sepsis			
Soft tissue infections	21/40 (53%)	21/40 (53%)	0.286
Perforated DU	7/40 (18%)	4/40 (10%)	
Empyema of the gall bladder	1/40 (3%)	6/40 (15%)	
Appendicular abscess	7/40 (18%)	5/40 (13%)	
Diverticulitis	2/40 (5%)	4/40 (10%)	
MVO	1/40 (3%)	0/40 (0%)	
Splenic abscess	1/40 (3%)	0/40 (0%)	
Duration of procedure (minutes)	76(48–89)	72(49–94)	> 0.999
EBL (ml)	400(300–600)	360(263–528)	0.449
Intraoperative fluids (ml)	1900(1450–2600)	2450 (1125–2800)	0.154
PaO2 / FiO2 (mmHg)	332(280–378)	319(269–379)	0.535
Platelet count (10^3^/mm^3^)	216(134–320)	179(126–327)	0.711
Bilirubin (mg/dl)	1.7(1.3–2.1)	1.4(0.8–2.1)	0.142
TLC (103/mm3)	18.5(16–23)	17(13.6–21.8)	0.084
CRP (mg/L)	239(153–318)	185(113–279)	0.078
HB (gm/dl)	12.5(11.2–14.1)	12.7(10.8–13.9)	0.950
Preoperative serum creatinine (mg/dl)	0.92(0.72–1.20)	0.89(0.82–1.10)	0.525
Patients with baseline creatinine > UNL, n	12/40 (30%)	7/40 (17.5%)	0.293

*BA* Bronchial asthma, BMI, Body mass index, *COPD* Chronic obstructive pulmonary disease, *CRP* C-reactive protein, *DM* Diabetes mellitus, *DU* Duodenal ulcer, *EBL* Estimated blood loss, *HB* Hemoglobin, *HR* Heart rate, *HTN* Hypertension, *MAP* Mean arterial pressure, *MVO* Mesenteric vascular occlusion, *SLE* Systemic lupus erythematosus, *SOFA* Sequential organ failure assessment, *TLC* Total leucocytic count, *UNL *Upper normal limit

**Fig. 2:**
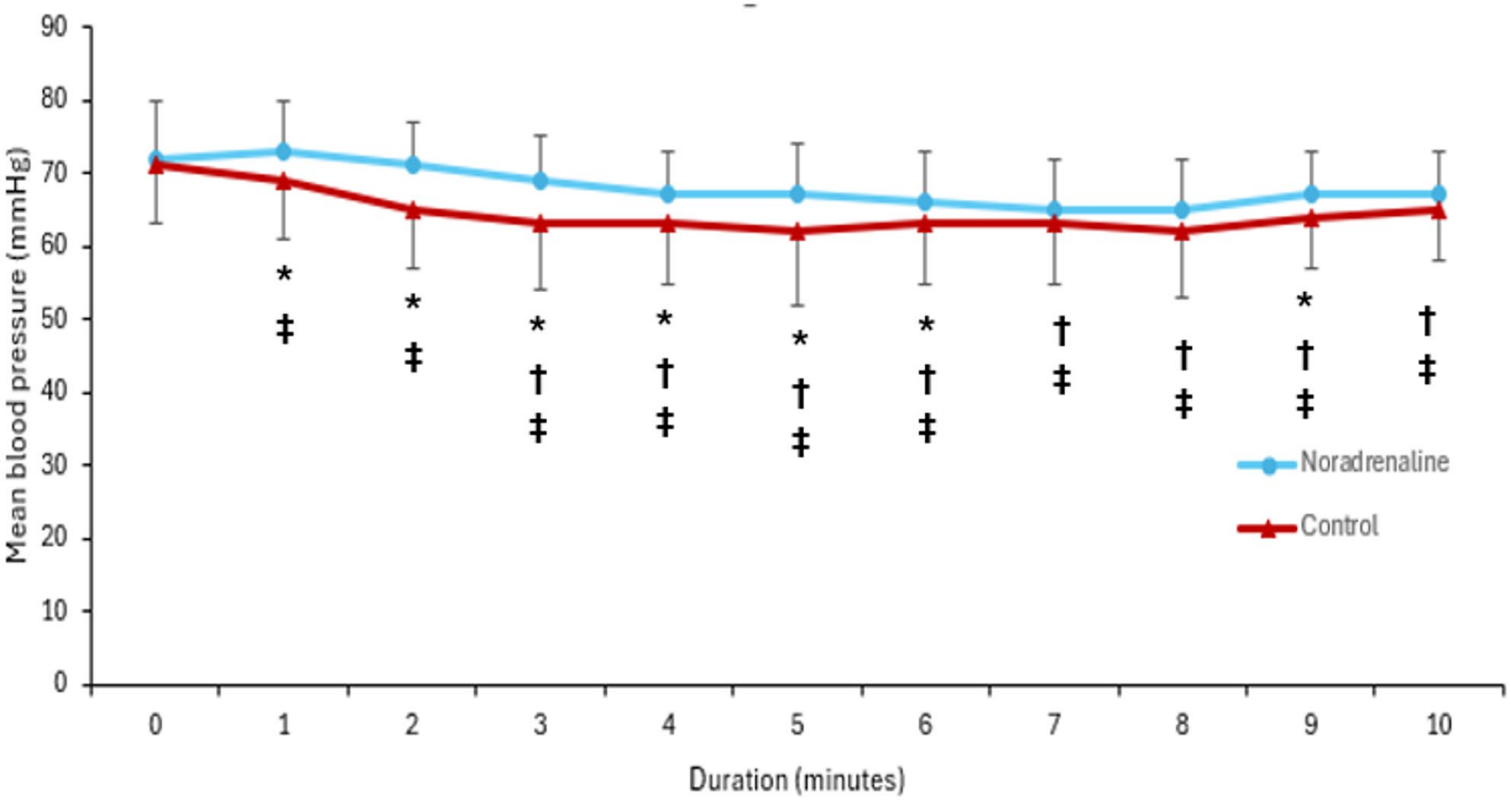
Mean blood pressure over time. *: Denotes significance in comparison to the other group. †: Denotes significance compared to baseline for noradrenaline group. ‡: Denotes significance compared to baseline for control group

**Fig. 3:**
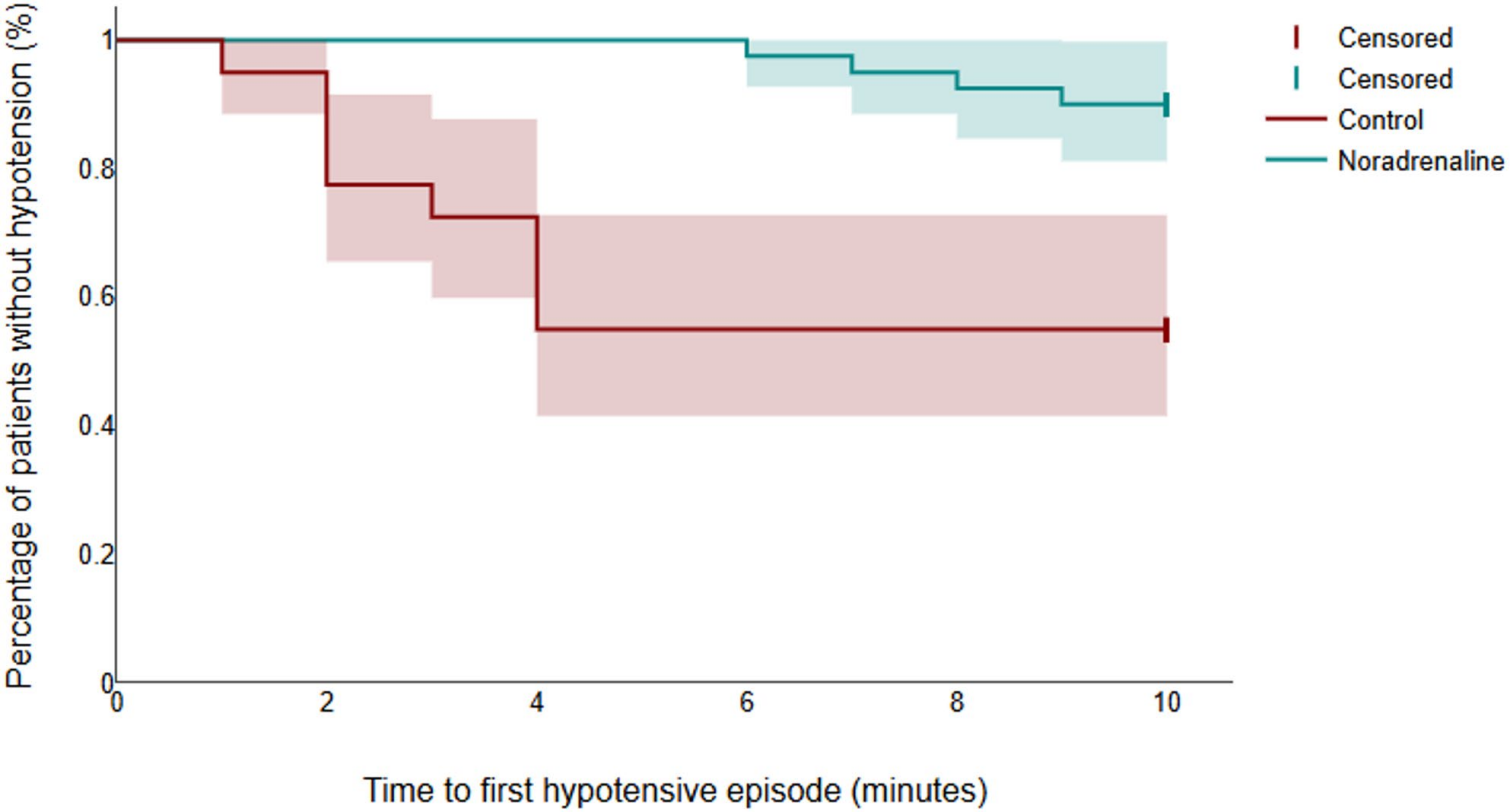
Analysis of time to first hypotensive episode as a secondary outcome using Kaplan–Meier curve, shaded areas represent 95% confidence interval

Laboratory parameters, including PaO₂/FiO₂ ratios, platelet counts, bilirubin levels, total leukocytic count (TLC), C-reactive protein (CRP), hemoglobin concentration, and preoperative serum creatinine, showed no statistically significant differences between groups (Table 1). However, the patient-related outcomes revealed several notable distinctions. MAP declined in both groups over the first 10 min, with a more pronounced reduction observed in the control group (Fig. 2). Notably, the time to the first hypotensive episode was significantly longer in the noradrenaline group (Table 2; Fig. 3). The incidence of hypotension was markedly lower in the noradrenaline group (10%) than in the control group (45%), and no cases of severe hypotension occurred in the noradrenaline group, whereas 13% of patients in the control group experienced severe episodes. The noradrenaline group also had fewer hypotensive episodes and required significantly lower doses of ephedrine.

**Table 2:** Intraoperative and postoperative outcomes, data presented as median (quartiles), and count (frequency)

	Noradrenaline group (*n* = 40)	Control group (*n* = 40)	Unadjusted *P* value	Adjusted *P* value
Incidence of hypotension, *n*	4/40 (10%)	18/40 (45%)	< 0.001*	-------
Incidence of severe hypotension, *n*	0/40 (0%)	5/40 (13%)	0.021*	0.105
Number of hypotensive episodes			0.005*	0.025*
• One	2/40 (5%)	6/40 (15%)		
• Two	2/40 (5%)	9/40 (23%)		
• Three	0/40 (0%)	3/40 (8%)		
Time to first episode (minutes)	7.5(6.3–8.8)	2.5(2–4)	< 0.001*	0.005*
Ephedrine requirements (mg)	12(7.5–16.5)	18(12-31.5)	0.002*	0.01*
Maximum postoperative creatinine (mg/dl)	1.16(0.90–1.30)	1.11(0.90–1.42)	0.424	> 0.999
Percentage of increase in creatinine (%)	8.5(3.4–20.2)	14(7.5–26.9)	0.072	0.432
Need for additional noradrenaline infusion, *n*	0/40 (0%)	3/40 (7.5%)	0.241	> 0.999
Incidence of AKI, *n*	2/40 (5%)	9/40 (23%)	0.048*	0.288
Incidence of MI, *n*	1/40 (3%)	2/40 (5%)	> 0.999	> 0.999
Need for RRT, *n*	0/40 (0%)	3/40 (8%)	0.241	> 0.999
Incidence of stroke, *n*	0/40 (0%)	2/40 (5%)	0.494	> 0.999
Incidence of delirium, *n*	1/40 (3%)	1/40 (3%)	> 0.999	> 0.999

*AKI* Acute kidney injury, *MI* Myocardial infarction, *RRT* Renal replacement therapy

*: Denotes statistical significance

Furthermore, the incidence of AKI was significantly lower in the noradrenaline group (5%) than in the control group (23%) (Table 2). Other outcomes, including myocardial infarction, need for RRT, stroke, and delirium, did not differ significantly between the groups. Overall, noradrenaline use was associated with improved hemodynamic stability and a reduced risk of AKI. Notably, 3 out of 40 patients in the control group required noradrenaline infusion within the range of 0.2–0.4 µg/kg/min after the 10-minute post-induction period.

## Discussion

This randomized controlled trial demonstrated that prophylactic administration of noradrenaline before induction of anesthesia significantly reduced the incidence and severity of PIH in patients undergoing source control surgery for sepsis. Despite comparable baseline characteristics, laboratory profiles, and intraoperative variables between the noradrenaline and placebo normal saline control groups, the noradrenaline group exhibited markedly improved hemodynamic stability. Specifically, the incidence of hypotension was reduced from 45% in the control group to 10% in the noradrenaline group, with no cases of severe hypotension. These findings align with prior evidence from cardiac anesthesia, where noradrenaline has been shown to maintain vascular tone and prevent hypotensive episodes during high-risk procedures such as transcatheter aortic valve implantation [14]. 

The pathophysiology of PIH in septic patients is multifactorial, involving vasodilation, impaired vascular responsiveness, and myocardial depression [1, 4]. Noradrenaline, a potent α-adrenergic agonist with mild β-activity, counteracts these effects by increasing systemic vascular resistance and stabilizing blood pressure. The delayed onset of hypotension and reduced ephedrine requirements in the norepinephrine group further support its role in maintaining peri-induction hemodynamics. Importantly, the reduction in AKI from 23% to 5% suggests that improved perfusion during induction may have downstream benefits on organ function, particularly renal integrity, findings consistent with previous studies linking PIH to renal hypoperfusion and injury [17]. 

Other outcomes, such as myocardial infarction, stroke, and delirium, did not differ significantly between groups; however, the study was not powered to detect differences in these less frequent events. Nonetheless, the absence of adverse effects related to noradrenaline infusion reinforces its safety in this context. The use of Ketofol for induction is known for its hemodynamic neutrality [18], may have contributed to overall stability, but does not account for the intergroup differences observed.

This study fills a critical gap in the literature by evaluating noradrenaline prophylaxis specifically in patients with sepsis, a population uniquely vulnerable to PIH. These findings suggest that early vasopressor support is a simple yet effective strategy for mitigating peri-induction risks.

It should be noted that only two perioperative renal protection strategies have been formally established to date: the KDIGO bundle, which emphasizes hemodynamic optimization, avoidance of nephrotoxins, and careful fluid management [19], and the 2024 EACTS/EACTAIC/EBCP Guidelines on cardiopulmonary bypass in adult cardiac surgery [20]. Our findings suggest that prophylactic noradrenaline infusion may complement these strategies by reducing the incidence of PIH and thereby mitigating renal hypoperfusion, although it should not be considered a direct renal protection intervention.

In line with our findings, a recent randomized controlled trial demonstrated that prophylactic noradrenaline infusion significantly reduced the incidence of hypotensive episodes in elderly patients receiving propofol infusion after spinal anesthesia [21]. Furthermore, regarding the method of vasopressor administration, a recent study showed that intermittent manual boluses were comparable to continuous infusion in maintaining hemodynamic stability [22]. 

Moreover, in patients undergoing transcatheter aortic valve implantation, prophylactic administration of noradrenaline has been shown to prevent PIH effectively [14]., supporting its role in maintaining perioperative hemodynamic stability in high-risk populations. To the best of our knowledge, this is the first randomized controlled trial to investigate the use of prophylactic noradrenaline specifically in patients with surgical sepsis, addressing a critical gap in the existing literature.

## Limitations

This study has several limitations that should be acknowledged. First, it was conducted at a single tertiary care center, which may limit the generalizability of the findings to other settings with different patient populations or clinical practices. Second, although the sample size was adequately powered to detect differences in the incidence of post-induction hypotension, it may have been insufficient to detect differences in less frequent secondary outcomes, such as myocardial infarction and stroke. Third, the study focused exclusively on short-term perioperative outcomes, and the long-term effects of noradrenaline administration, such as renal recovery or neurocognitive sequelae, were not assessed. Fourth, the use of Ketofol as the induction agent, although standardized across groups, may not reflect common practice in all institutions and could influence hemodynamic responses differently than other agents. Fifth, Although patients with renal impairment, defined as baseline serum creatinine > 1.5 × the UNL (UNL; >1.8 mg/dL in males and > 1.5 mg/dL in females), were excluded from the study, patients with baseline creatinine values above the UNL but less than 1.5 × UNL were eligible for inclusion, and their distribution was reported to provide transparency. In addition, the definition of AKI applied in this study was based on either an increase in serum creatinine > 0.3 mg/dL or > 1.5-fold from baseline, assessed over a 7‑day postoperative window rather than the conventional 48‑hour period. Sixth, the study did not include invasive hemodynamic monitoring, which could have provided more precise insights into the changes in cardiac output and systemic vascular resistance following noradrenaline administration. Finally, arrhythmias were not systematically recorded as predefined outcomes, which may limit the assessment of potential electrophysiological effects of noradrenaline.

## Conclusion

In patients with sepsis undergoing source control surgery, the use of prophylactic noradrenaline (0.2 µg/kg/min) 5 min before induction of anesthesia was associated with significantly improved hemodynamic outcomes. Compared to the control group, patients receiving noradrenaline experienced a markedly lower incidence of post-induction hypotension (10% vs. 45%), and no cases of severe hypotension were observed. The time to the first hypotensive episode was significantly delayed, and the total number of episodes was reduced. Additionally, the noradrenaline group required lower doses of ephedrine to maintain the target blood pressure than the norepinephrine group. While the incidence of AKI was lower in the noradrenaline group, this secondary outcome should be interpreted cautiously, given the study’s limited power and multiple comparisons. The primary conclusion remains that prophylactic noradrenaline significantly reduces PIH and appears safe without increasing organ dysfunction. 

## Data Availability

The datasets used for analysis and/or generation in this study are available from the corresponding author upon reasonable request.
